# Oleylphosphocholine (OlPC) arrests *Cryptosporidium parvum* growth *in vitro* and prevents lethal infection in interferon gamma receptor knock-out mice

**DOI:** 10.3389/fmicb.2015.00973

**Published:** 2015-09-23

**Authors:** Karine Sonzogni-Desautels, Axel E. Renteria, Fabio V. Camargo, Thomas Z. Di Lenardo, Alexandre Mikhail, Michael J. Arrowood, Anny Fortin, Momar Ndao

**Affiliations:** ^1^National Reference Centre for Parasitology, Research Institute of the McGill University Health Centre, MontrealQC, Canada; ^2^Institute of Parasitology, Macdonald Campus, McGill University, MontrealQC, Canada; ^3^Department of Experimental Medicine, McGill University, MontrealQC, Canada; ^4^Division of Foodborne, Waterborne, and Environmental Diseases, Center for Disease Control and Prevention, AtlantaGA, USA; ^5^Department of Biochemistry, McGill University, MontrealQC, Canada; ^6^Dafra Pharma R&DTurnhout, Belgium

**Keywords:** *Cryptosporidium parvum*, cryptosporidiosis, oleylphosphocholine, OlPC, miltefosine, interferon gamma receptor knockout mice, oocysts, parasite burden

## Abstract

*Cryptosporidium parvum* is a species of protozoa that causes cryptosporidiosis, an intestinal disease affecting many mammals including humans. Typically, in healthy individuals, cryptosporidiosis is a self-limiting disease. However, *C. parvum* can cause a severe and persistent infection that can be life-threatening for immunocompromised individuals, such as AIDS patients. As there are no available treatments for these patients that can cure the disease, there is an urgent need to identify treatment options. We tested the anti-parasitic activity of the alkylphosphocholine oleylphosphocholine (OlPC), an analog of miltefosine, against *C. parvum* in *in vitro* and *in vivo* studies. *In vitro* experiments using *C. parvum* infected human ileocecal adenocarcinoma cells (HCT-8 cells) showed that OlPC has an EC_50_ of 18.84 nM. Moreover, no cell toxicity has been seen at concentrations ≤50 μM. C57BL/6 interferon gamma receptor knock-out mice, were infected by gavage with 4000 *C. parvum* oocysts on Day 0. Oral treatments, with OlPC, miltefosine, paromomycin or PBS, began on Day 3 post-infection for 10 days. Treatment with OlPC, at 40 mg/kg/day resulted in 100% survival, complete clearance of parasite in stools and a 99.9% parasite burden reduction in the intestines at Day 30. Doses of 30 and 20 mg/kg/day also demonstrated an increased survival rate and a dose-dependent parasite burden reduction. Mice treated with 10 mg/kg/day of miltefosine resulted in 50% survival at Day 30. In contrast, control mice, treated with PBS or 100 mg/kg/day of paromomycin, died or had to be euthanized between Days 6 and 13 due to severe illness. Results of parasite burden were obtained by qPCR and cross-validated by both flow cytometry of stool oocysts and histological sections of the ileum. Together, our results strongly support that OlPC represents a potential candidate for the treatment of *C. parvum* infections in immunocompromised patients.

## Introduction

*Cryptosporidium parvum* is an obligate parasite of the phylum Apicomplexa that infects the microvilli of the small intestine of many mammalian hosts including humans (Ryan and Xiao, 2014). Considered as a major waterborne pathogen (drinking and recreational water) that can also be transmitted through contaminated foods (Fayer et al., 2000; Cacciò and Putignani, 2014), *C. parvum* outbreaks have been widely reported in numerous countries from all continents (Cacciò et al., 2005; Putignani and Menichella, 2010; Cacciò and Putignani, 2014). This parasite can remain infectious for months in a humid environment (Robertson et al., 1992; King et al., 2005; Cacciò and Putignani, 2014). As a result, *C. parvum* has been categorized as a class B bioterrorist pathogen by the Center for Disease Control and Prevention (CDC; Feldmann et al., 2002; Rotz et al., 2002).

As one of the major causative agents of cryptosporidiosis in humans, along with *Cryptosporidium hominis* (Cacciò et al., 2005), *C. parvum* causes self-limiting watery diarrhea or persistent and severe diarrhea depending on the age and the immune status of the patient (Chalmers and Davies, 2010; Putignani and Menichella, 2010; Shikani and Weiss, 2014). Indeed, *C. parvum* infection has been reported to be life-threatening in AIDS patients, among which the prevalence of cryptosporidiosis was determined to be 14% in developed countries and 24% in developing countries (Collinet-Adler and Ward, 2010; Putignani and Menichella, 2010). *Cryptosporidium* spp., along with *Giardia* spp., has also been identified as the leading cause of chronic or persistent diarrhea in children in a context of malnutrition or immunodeficiency (Thapar and Sanderson, 2004; Putignani and Menichella, 2010). The Global Enteric Multicenter Study identified Cryptosporidium as one of the top five pathogens causing mild to severe diarrhea in children under the age of 2 in developing countries (Kotloff et al., 2013). Studies have shown that *Cryptosporidium* infections in young children have often resulted in stunting and lead to poor cognitive functions later in childhood (Berkman et al., 2002). However, currently available treatments have demonstrated limited effect in these vulnerable populations (Mead and Arrowood, 2014).

Nitazoxanide, the only American Food and Drug Association (FDA) approved drug to treat cryptosporidiosis in immunocompetent patients, has shown little activity to fight against *C. parvum* infections in AIDS patients (Cabada and White, 2010; Rossignol, 2010; Mead and Arrowood, 2014). Similarly, paromomycin, the currently used drug to treat *C. parvum* infections in AIDS patients, has shown modest activity and limited results in different case studies (Hewitt et al., 2000; Rossignol, 2010). With no efficient way to treat immunocompromised patients, many drugs have been tested over the years, but very few have shown consistent activity (Mead and Arrowood, 2014).

Oleylphosphocholine (OlPC, C23H48NO4P) belongs to the family of alkylphosphocholines (Hernandez et al., 2014) and is structurally related to miltefosine (van Blitterswijk and Verheij, 2008), with the chemical formula C21H46NO4P ⋅ xH2O. Both compounds have shown *in vitro* and *in vivo* anti-leishmanial activity (Fortin et al., 2014), and *in vitro* activity of miltefosine against *C. parvum* has also been reported (Shahiduzzaman et al., 2009). This study reports the strong activity of OlPC, both *in vitro* and *in vivo* in an immunocompromised C57BL/6 IFNγR-KO mouse model of *C. parvum* infection.

## Materials and Methods

### Parasites

*Cryptosporidium parvum* (Iowa strain) oocysts, generously provided by Dr. Michael J. Arrowood (CDC, Atlanta, GA, USA), were maintained through a C57BL/6 IFNγR-KO mouse infection model previously described by our laboratory (von Oettingen et al., 2008). Oocysts were purified following the reported technique (von Oettingen et al., 2008) and were stored in 2.5% (w/v) aqueous potassium dichromate (K_2_Cr_2_O_7_, Sigma-Aldrich, Oakville, ON, Canada) at 4°C.

### Test Compound

Oleylphosphocholine was generously provided by Dafra Pharma Research and Development (Turnhout, Belgium) and stored at 4°C, away from light. For *in vitro* studies, OlPC was diluted to a stock solution of 11.5 mM in ddH_2_O. Miltefosine (Sigma-Aldrich, Oakville, ON, Canada) was used as a control in the *in vitro* assays because of its previously reported activity against *C. parvum* (Shahiduzzaman et al., 2009). Paromomycin (Sigma-Aldrich) was also used as a control in the *in vitro* assays. The drugs were prepared fresh daily in phosphate-buffered saline (PBS) for animal studies.

### Host Cells

For *in vitro* studies, human colonic tumor cells (ileocecal adenocarcinoma; HCT-8: ATCC CCL-244) were used. Cells were maintained in T225 flasks (BD Bioscience, Mississauga, ON, Canada) using DMEM media (Wisent, St-Bruno, QC, Canada). For OlPC *in vitro* studies, media was supplemented with 10 μg/ml gentamycin, 10 μg/ml streptomycin, 100 U/ml penicillin, 10% heat-inactivated fetal bovine serum (FBS), and non-essential amino acids (NEAA; Wisent). For the cytotoxicity assays, cells were grown in supplemented DMEM without phenol red and further supplemented with 4.5 g/L D-glucose (Sigma-Aldrich, Oakville, ON, Canada) and 110 mg/L sodium pyruvate (Sigma-Aldrich). For miltefosine and paromomycin *in vitro* studies, media was supplemented with 10 μg/ml gentamicin, 50 μg/ml streptomycin, 50 U/ml penicillin, and 10% heat-inactivated FBS.

### Cytotoxicity of OlPC in HCT-8 Cells

XTT assay (Sigma-Aldrich) was used (Roehm et al., 1991), which relies on the ability of live cells to cleave XTT causing a colorimetric change, to quantify the possible cytotoxicity of OlPC in HCT-8 cells. For this assay, DMEM without phenol red was used. Initially, 5 × 10^4^ HCT-8 cells in 200 μL of supplemented DMEM were seeded in a 96-well flat-bottom plates (BD Falcon, Franklin Lakes, NJ, USA) and incubated overnight (or until they reached ∼80% confluency) at 37°C with 5% CO_2_. Freshly prepared serial dilutions of OlPC diluted in supplemented DMEM were used and 50 μL of these solutions were added to the wells to obtain final OlPC concentrations ranging from 10 to 1000 μM (4.33 to 433 μg/mL). Samples were run in quadruplicate and plates were incubated at 37°C with 5% CO_2_ for 48 h. Then, 50 μL of XTT (dissolved in DMEM) was added to each well and plates were re-incubated for 4 h. Absorbance was measured at 450 nm using a plate reader (EL800 BioTek; Fisher, Nepean, ON, Canada).

### *C. parvum* Infection Model in HCT-8 Cells

We tested the effect of OlPC on *C. parvum* sporozoites in HCT-8 cells. Briefly, 24-well flat-bottom plates (BD Falcon) were seeded with 3 × 10^5^ cells in 1.4 mL of supplemented DMEM and incubated overnight (or until they reached ∼80% confluency) at 37°C with 5% CO_2_. Stock oocysts in 2.5% K_2_Cr_2_O_7_ were washed three times with 0.1 M acetate-NaCl buffer, pH 5.5, and subsequently incubated in 10 mM sodium periodate on ice for 20 min. Oocysts were then washed three times with PBS containing 0.1% bovine serum albumin (BSA; Sigma-Aldrich). To release the sporozoites from the oocysts (excystation), parasites were incubated in DMEM containing 0.75% sodium taurocholate (Sigma-Aldrich) at 37°C for 30 min or until 50% excystation was determined by microscopy. Then, sporozoites from 1 × 10^5^ oocysts were inoculated in each well. Non-infected cells incubated with supplemented DMEM served as negative controls as well as infected cells incubated with supplemented DMEM, as positive controls. OlPC was serially diluted in supplemented DMEM and was subsequently added to each well at concentrations ranging from 10 pM to 100 μM (4.33 pg/mL to 43.3 μg/mL). Miltefosine and paromomycin, diluted in supplemented DMEM, were also tested at concentrations ranging from 10 pM to 10 μM (4.08 pg/mL to 4.08 μg/mL) and 10 pM to 100 μM (7.14 pg/mL to 71.4 μg/mL) respectively, to serve as controls. Plates were further incubated for 48 h at 37°C with 5% CO_2_. *In vitro* assays were run two independent times and each condition was run in triplicate for the miltefosine assay and in duplicate for the OlPC and the paromomycin assays. At the completion of the incubation, supernatants were removed and cells were lifted using 0.25% trypsin in EDTA (Wisent). To assess parasite burden in each well and determine the efficacy of OlPC, miltefosine or paromomycin against *C. parvum*, DNA was extracted from samples using QIAamp DNA Mini Kit (Qiagen, Toronto, ON, Canada). Purified DNA was stored at -20°C until use.

### Quantitative Polymerase Chain Reaction Primers, Probe, and Standard Curve

Parasite burden from DNA samples from *in vitro* assays as well as from mouse fecal and intestinal samples were assessed by quantitative polymerase chain reaction (qPCR) targeting the hsp70 gene of *C. parvum* (GenBank: U69698.2) using the LightCycler 2.0 system (Roche, Laval, QC, Canada). Primers and probe sequences (TaqMan) were used according to previously published sequences (Shahiduzzaman et al., 2009). Briefly, a master mix per sample was prepared by adding 5 μL of PCR-grade water (Roche), 2 μL of 10 μM of forward primer (CP_hsp70_fwd: 5′-AACTTTAgCTCCAgTTgAgAAAgTACTC-3′; Tib MolBiol, Adelphia, NJ, USA), 2 μL of 10 μM reverse primer (CP_hsp70_rvs: 5′-CATggCTCTTTACCgTTAAAgAATTCC-3′; MolBiol), 2 μL of 2 μM 5′ 6-carboxyfluorescein reporter dye (FAM) probe with a 3′ tetramethylrhodamine quencher dye (TMR; hsp-taq: 5′-AATACgTgTAgAACCACCAACCAATACAACATC-3′; MolBiol), and 4 μL of buffer mix containing dNTP, MgCl_2_, and Taq-polymerase (Roche). Prior to the polymerase chain reaction, 5 μL of sample and 15 μL of master mix were added to LightCycler capillaries (Roche). The initial denaturation step was conducted at 95°C for 15 min followed by 45 cycles of denaturation at 94°C for 15 s and annealing at 60°C for 1 min (Shahiduzzaman et al., 2009).

In order to quantify parasite burden, a standard curve was necessary. First, *C. parvum* oocysts were diluted to concentrations of 1 × 10^1^ to 1 × 10^6^ in 200 μL and then, they were disrupted by five freeze-thaw cycles of 2 min incubation in liquid nitrogen followed by 2 min incubation in a 56°C water bath. Second, DNA from the oocyst extracts was purified using QIAamp DNA Blood Mini Kit (Qiagen) and these samples were run (qPCR) in the LightCycler 2.0; qPCR was used to correlate threshold cycle values (C_t_ values) to parasite burden (*R*^2^ = 0.9989). This standard curve was used to extrapolate parasite burden from DNA samples from *in vitro* assays as well as from mouse stool and intestine samples.

### Immunocompromised Mouse Model of *C. parvum* Infection

*Cryptosporidium parvum* oocysts in 2.5% K_2_Cr_2_O_7_ were washed three times with PBS; parasites were then counted using a hemocytometer and diluted in PBS to a concentration of 4 × 10^4^/mL. Six to eight week old C57BL/6 IFNγR-KO female and male mice (Jackson Laboratories, Bar Harbor, ME, USA) were infected with a lethal dose of 4000 oocysts in 100 μL of PBS on Day 0 using a 22-gage gavage needle (CDVM, St-Hyacinthe, QC, Canada). Mice were separated in eight groups (13 mice/group for treated groups and 4–8 mice/group for control groups). Groups 1 and 2 were non-infected controls, treated per os (oral gavage) with PBS or with 40 mg/kg/day of OlPC, respectively. Group 3 was infected and treated with PBS (positive controls). Groups 4–6 were infected and treated per os with 40, 30, and 20 mg/kg/day of OlPC, respectively. Groups 7 and 8 were infected controls treated per os with 10 mg/kg/day of miltefosine and 100 mg/kg/day of paromomycin, respectively. Treatment started at Day 3 and continued for 10 days. OlPC and control drugs were prepared fresh on a daily basis for the length of the treatment. Mice were weighted daily. Starting on Day 4, individual stool samples were collected every 3–4 days until study termination.

Five mice from each group were sacrificed at Day 10 (before daily treatment) to assess and compare disease progression. Other mice were maintained until study termination (Day 30) unless they were found dead or had to be sacrificed due to severe illness. Because of the lethality of this model (von Oettingen et al., 2008; Ndao et al., 2013) an objective mouse rating system based on symptom severity and behavioral changes was developed in accordance with the Animal Care Committee and the Canadian Council on Animal Care (CCAC) guidelines to determine when mice should be sacrificed due to illness. Upon death or euthanasia, the mouse ileum was preserved in formalin for histological purposes and the rest of the intestine (duodenum to rectum) was collected individually and chopped into 0.5 cm pieces into a 60 mL specimen container (ThermoFisher Scientific, Ottawa, ON, Canada) with 10 mL of 0.02% (v/v) Tween20 in PBS (one container per mouse). During necropsy, stool was also collected for oocyst purification.

After completion of the *in vivo* study, a constant volume of purified oocysts from intestinal samples of mice from each treatment groups was inoculated to naïve C57BL/6 IFNγR-KO mice to determine if the concentration of oocysts remaining in the intestines of treated mice was able to induce clinical signs of cryptosporidiosis in naïve mice.

All mouse studies were conducted at the Montreal General Hospital rodent animal facility site of the Research Institute of McGill University Health Center. All studies performed were approved and in accordance with the MGH Facility Animal Care Committee and also in accordance with the CCAC guidelines.

### Oocyst Purification from Intestinal Samples and Oocyst Burden Analysis by qPCR

For each mouse intestine sample in 0.02% (v/v) Tween20 in PBS, 0.05 g of sputasol (dry mixture of 10% DTT, 76% NaCl, 2% KCl, 10% Na_2_HPO_4_, and 2% KHPO_4_) was added. Oocyst purification was performed using a previously described method reported in literature and by our lab (Meloni, 1996; von Oettingen et al., 2008). Briefly, each sample was homogenized for 2 min at 7000 rpm with the Polytron PT3000D homogenizer (Kinematica, Lucerne, Switzerland). Between each sample, the homogenizer was cleaned with bleach and ethanol to avoid cross-contamination. Samples were placed on a rotary mixer at low speeds for 90 min at room temperature. Samples were then transferred to 50 mL Falcon tubes (BD Falcon, Franklin Lakes, NJ, USA) and centrifuged at 2000 × *g* for 10 min at 4°C. The supernatant was removed and the pellet was resuspended in 8 mL of 0.02% (v/v) Tween20 in ddH_2_O and 2 mL of diethyl ether (Sigma-Aldrich, Oakville, ON, Canada). Samples were thoroughly vortexed and centrifuged at 2000 × *g* for 10 min at 4°C. The layers containing fatty cells and tissues as well as the supernatants were removed. The pellets were washed with 20 mL of cold ddH_2_O and centrifuged at 2000 × *g* for 10 min at 4°C. Supernatants were discarded and the pellets resuspended in 20 mL of saturated NaCl. Samples were vortexed thoroughly, carefully overlayed with 5 mL of cold ddH_2_0 and centrifuged at 2000 × *g* for 10 min at 4°C. Five milliliters of the interphase were collected and centrifuged at 2000 × *g* for 10 min at 4°C; supernatants were discarded and oocyst-containing pellets were resuspended in 1 mL PBS containing 10 μg/ml streptomycin and 100 U/ml penicillin. Purified oocyst samples were stored at 4°C. For qPCR analysis, 200 μL were taken from each purified oocyst sample and five freeze/thaw cycles of 2 min incubation in liquid nitrogen followed by 2 min incubation in a 56°C water bath were performed. DNA was then extracted using the QIAamp DNA Blood Mini Kit (Qiagen) and samples was used for qPCR analysis.

### Oocyst Purification from Stool Samples and Oocyst Shedding Analysis by qPCR

Individual stool samples were collected in 500 μL of distilled and deionized (18.2 MΩ cm) water (ddH_2_O) and stored in microcentrifuge tubes at 4°C until used. Samples were thoroughly vortexed until stools were homogenized to smaller particles, then centrifuged at 14000 × *g* and washed with cold ddH_2_O. Supernatants were discarded and the pellets resuspended in 1 mL of saturated NaCl. Samples were vortexed thoroughly, carefully overlayed with 250 μL of cold ddH_2_O and centrifuged at 1600 × *g* for 10 min at 4°C. All of the supernatant was collected and the sample was exposed to another NaCl-ddH_2_O overlay. The supernatants collected from both overlays were centrifuged at 14000 × *g* for 3 min at 4°C, combined into one tube and resuspended in 200 μL of ddH_2_O. Purified oocysts were exposed to five freeze/thaw cycles as described above. DNA was extracted using the QIAamp DNA Blood Mini Kit and stored at -20°C until oocyst shedding was quantified by qPCR. Values were normalized by gram of stool.

### Oocyst Burden Analysis by Flow Cytometry

All oocyst samples purified from mouse intestine were incubated in a final concentration of 1% paraformaldehyde for 15–30 min prior to analysis. Briefly, 40 ul of sample and 50 ul of CountBright^TM^ absolute counting beads (Invitrogen, Burlington, ON, Canada) were added to a paraformaldehyde solution for a final volume of 500 ul. Oocysts within each sample were quantified by morphology (FSC-A and SSC-A) using BD LSRFortessa^TM^ cell analyser (BD, Franklin Lakes, NJ, USA). Oocysts were initially identified with a FITC-labeled mouse IgG3 monoclonal antibody targeting a surface antigen (AbD Serotec, Raleigh, NC, USA). Results demonstrated that 98% of the FITC positive events occupied a distinct population by size and complexity in the FSC-A and SSC-A channels. Results generated by flow cytometry are based on unstained samples gating on this distinct oocyst population. Oocyst concentration per sample was calculated by determining the quantity of sample collected based on the number of CountBright^TM^ bead events collected. Final oocyst numbers were normalized by gram of mouse intestines.

### Histopathology

*Cryptosporidium parvum* is known to preferentially infect the jejunum and ileum (Fayer and Xiao, 2008), thus the ileum was collected for histopathology. Liver and spleen were also fixed in formalin, paraffin-embedded and cut to 4 μm thick sections. Sections were stained with hematoxylin and eosin to further assess disease progression by light microscopy.

### Data Analysis

All *in vitro* studies and qPCR data were analyzed using Microsoft Excel (Microsoft Corporation, Redmond, WA, USA). Graphs and *P*-values were obtained using GraphPad Prism^®^ version 6.0 (GraphPad Software, Inc., La Jolla, CA, USA). Results represent mean ± standard error mean (SEM). For OlPC and miltefosine EC_50_ calculations, the same software, GraphPad Prism^®^ version 6.0, the non-linear fit of EC_50_ shift and the “shared value for all data sets” constraint for Bottom and Top were used. Flow cytometry data was analyzed using FlowJo v10 analytical software (Treestar, Inc., San Carlos, CA, USA).

## Results

### OlPC and Miltefosine Inhibit *C. parvum* Infection in HCT-8 Cells *In Vitro*

To test the efficacy of OlPC to inhibit *C. parvum* infection *in vitro*, we used HCT-8 cells which allowed the parasite to replicate (Widmer et al., 2000). Miltefosine was used as a control as its efficiency to inhibit *C. parvum* infection *in vitro* has already been described (Shahiduzzaman et al., 2009). However, the dose of 100 μM was not tested for miltefosine as cell toxicity has been reported at a concentration of 24.5 μM when cells are incubated for 45 h (Shahiduzzaman et al., 2009). For the OlPC and the miltefosine assays, DNA was extracted after 48 h of incubation with the compound. A standard curve was used to correlate threshold cycle values (C_t_ values) obtained by qPCR to parasite burden. Then, for each well, the percentage of parasite burden reduction was assessed according to this formula:

=[1-(parasite burden from tested well/parasite burden of positive control)]^∗^100

**FIGURE 1:**
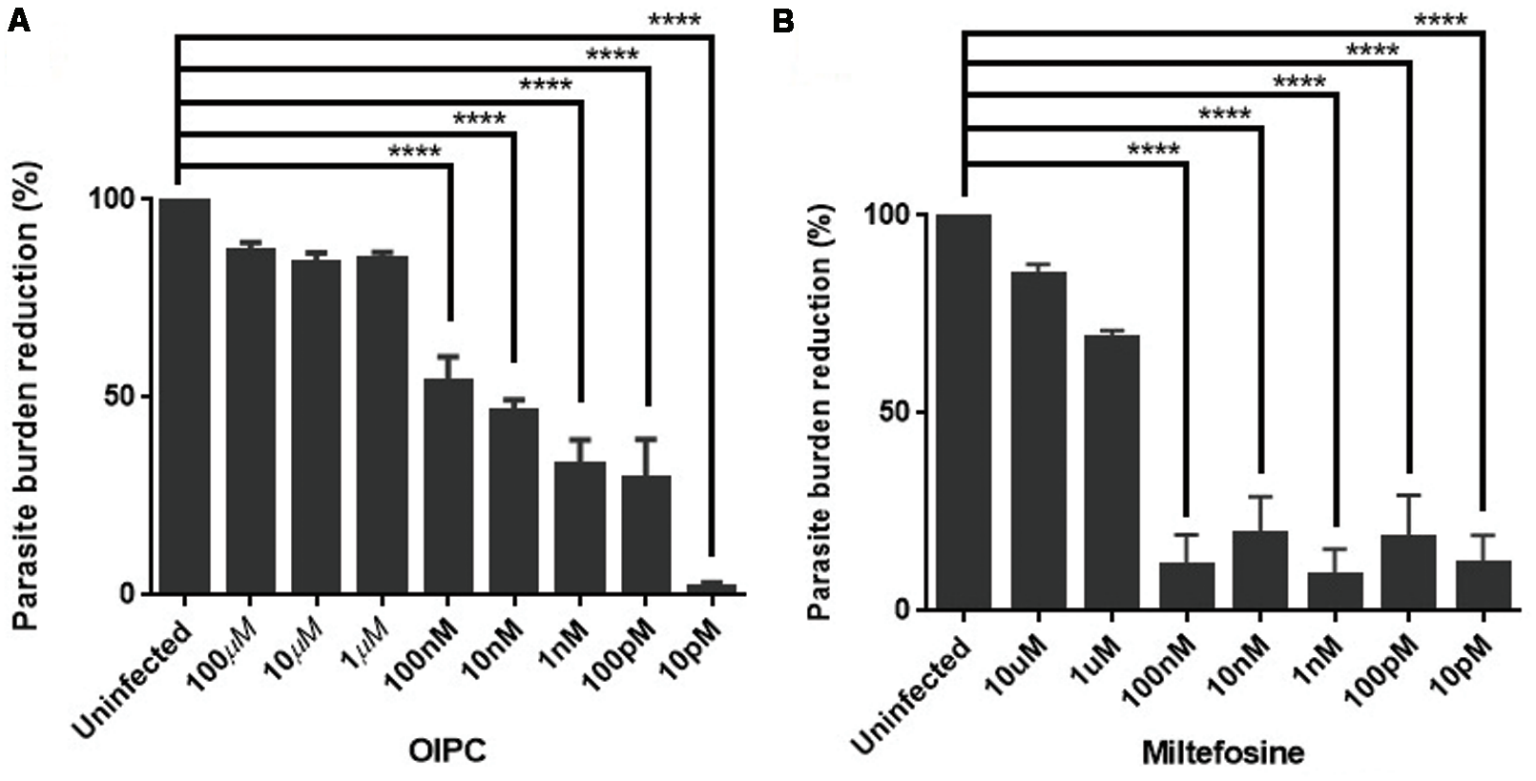
**Efficacy of OlPC and miltefosine *in vitro*. (A)** Parasite burden reduction in HCT-8 cells after 48 h incubation with different concentrations of OlPC. **(B)** Parasite burden reduction in HCT-8 cells after 48 h incubation with different concentrations of miltefosine. Error bars were calculated as ±SEM (^∗∗∗∗^*P* < 0.0001).

Positive controls were infected cells incubated with supplemented media (no compound). **Figure 1** represents the mean of the parasite burden reduction of tested wells for each concentration of OlPC (A) and miltefosine (B) ± SEM.

HCT-8 cells treated with 100, 10, and 1 μM of OlPC showed the highest *C. parvum* inhibition with 87, 84, and 85% reduction in parasite burden, respectively (**Figure 1A**). Therefore, these concentrations of OlPC are not statistically different than uninfected controls showing the clear potency of these treatments to inhibit *C. parvum* infection *in vitro*. There is a statistical difference (*P* < 0.0001) between OlPC treatment and uninfected controls starting at the concentration of 100 nM due to the lack of efficiency of these doses to inhibit *C. parvum* infection. Results demonstrated a dose-dependent reduction of *C. parvum* burden when treated with increasing concentrations of OlPC and a calculated EC_50_ of 18.84 nM. Cell toxicity of OlPC was not observed at concentrations of ≤50 μM using an XTT assay (data not shown).

Miltefosine at concentrations of 10 and 1 μM showed the highest inhibitory effect with 85 and 69% reduction in parasite burden, respectively (**Figure 1B**). Similarly to the OlPC treatment, at these concentrations, there is no statistical difference between the miltefosine treatment and uninfected cells. So, OlPC is as potent as miltefosine at inhibiting *C. parvum* infection *in vitro* at a concentration of 10 μM (84 vs. 85%), but OlPC better inhibits infection at a concentration of 1 μM (85 vs. 69%). The calculated EC_50_ for miltefosine is 0.81 μM, which is slightly lower than the previously published value of 1.87 μM for this compound and this parasite (Shahiduzzaman et al., 2009), but within the same range. Batch differences in oocyst virulence due primarily to freshness are normal and can explain this type of variation. Concentrations of paromomycin ranging from 10 pM to 100 μM were tested to determine the ability of this compound to inhibit *C. parvum* infection *in vitro*. However, no significant effect was observed (data not shown), which is in agreement with what has been published previously (Ndao et al., 2013).

### OlPC shows a Dose-Dependent Effect on *C. parvum*-Induced Mortality in an Immunodeficient Mouse Model

The C57BL/6 IFNγR-KO mouse model is an excellent model to assess the efficacy of compounds to treat acute *C. parvum* infection because of the high susceptibility of these knockout mice to this parasite (von Oettingen et al., 2008; Ndao et al., 2013). According to previous reports, mice infected with as low as 1500 oocysts had a mortality rate of 80% by Day 14 (Ndao et al., 2013). Therefore, in this study, mice were infected with 4000 oocysts, and survival, as well as parasite burden reduction, was used to differentiate an effective treatment from an inefficient treatment. To ensure the objectivity of the study, an external blinded examiner scored the mice reaching pre-defined critical clinical end-points according to a pre-established rating system.

**FIGURE 2:**
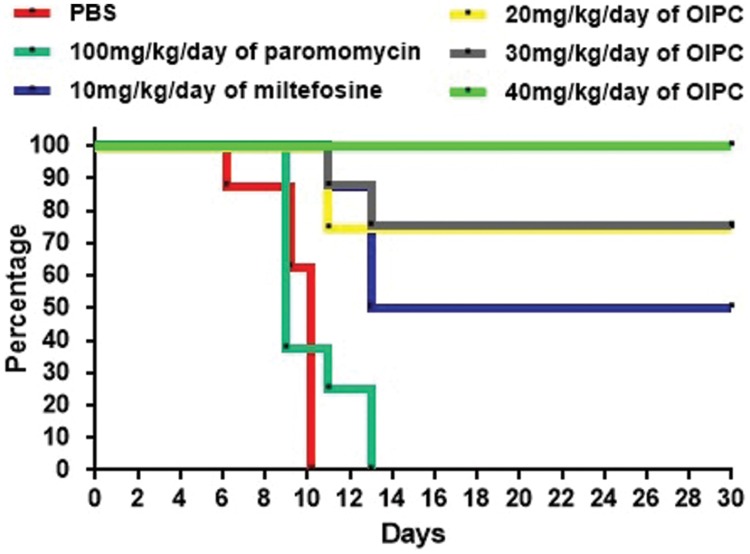
**Survival curves in C57BL/6 IFNγR-KO mice infected with *C. parvum*.** Results are shown in a survival curve. Results do not include mice sacrificed at Day 10 for disease progression analysis. On Day 30 remaining mice were sacrificed along with uninfected controls.

As expected, infected controls started showing symptoms of illness as early as Day 5 and had a very high mortality rate at Day 14. For the PBS treated group, mice started showing signs of illness (such as hunched back, weight loss, dehydration, lethargy, and watery stools) at Day 5 and all mice from this group died or were euthanized between Days 6 and 10 (**Figure 2**). Mice treated with 100 mg/kg/day of paromomycin for 10 days displayed clinical symptoms of cryptosporidiosis at Day 7 and a 60% mortality rate was observed at Day 9. Some paromomycin-treated mice did survive until Day 13 but, the Mantel-Cox or the Gehan–Breslow–Wilcoxon tests used to compare survival curves showed no significant difference with the PBS-treated group (**Figure 2**).

Mice treated with 10 mg/kg/day of miltefosine, the analog of OlPC, also showed signs of illness as early as Day 7 and a 50% mortality rate at Day 13 but, survivors remained alive until the end of the study (Day 30; **Figure 2**). This clear difference in survival rates with the PBS-treated group (*P* < 0.001) determined that a 10 mg/kg/day miltefosine treatment for 10 days significantly decreased mortality. Nevertheless, at Day 30, clinical signs of cryptosporidiosis were still visible in these mice such as 15% weight loss, hunched backs, dehydration, and soft stools.

Mice treated with 40 mg/kg/day of OlPC displayed a 100% survival rate by the experimental endpoint (Day 30; *P* < 0.001; **Figure 2**). Additionally, the onset of symptoms began later (Day 9) than the PBS-treated controls; clinical signs were less severe and did not last as long in comparison with other groups. Furthermore, at Day 10, which is the peak of infection (von Oettingen et al., 2008), weight loss (about 10% reduction from initial weight for the 40 mg/kg/day OlPC-treated group) was already significantly lower compared to any of the infected control groups (>15% of initial weight, *P* < 0.05, data not shown). By Day 30, 40 mg/kg/day OlPC-treated mice looked healthy as they displayed no visible signs of illness. Moreover, unlike the miltefosine-treated group, 40 mg/kg/day OlPC-treated mice had exceeded, by Day 30, their initial weights (*P* < 0.0001, data not shown). Mice treated with 30 mg/kg/day of OlPC for 10 days, had less severe clinical signs of cryptosporidiosis. For these latter mice, on one hand, treatment was not able to rescue all mice which showed a severe weight loss of up to 15% of initial weight; on the other hand, the onset of symptoms was delayed to Days 8 or 9. Their survival rate was of 87.5% at Day 11, 75% at Day 30 (**Figure 2**) and, by Day 30, the survivors displayed no visible signs of illness and had returned to their initial weights (data not shown). Finally, the lowest dose of OlPC (20 mg/kg/day) was still able to rescue 75% of mice at Day 30 even if there were still visible signs of illness and mice had a 5% weight loss in comparison with initial weights. Non-infected mice treated with 40 mg/kg/day of OlPC presented a constant weight through the length of the study and no clinical sign were noted in this group (data not shown).

### OlPC Eliminates Oocyst Shedding in a Dose-Dependent Manner

**FIGURE 3:**
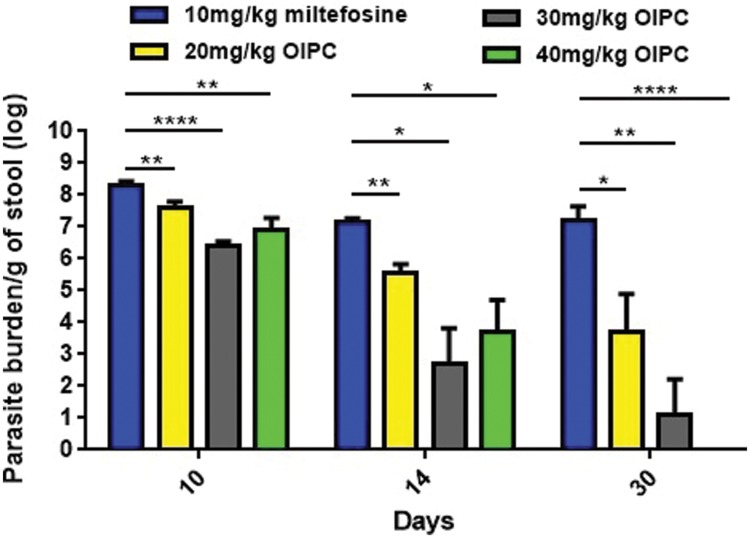
**Oocyst shedding in C57BL/6 IFNγR-KO mice infected with *C. parvum.*** All stool samples were collected from individual mice within each group, processed and analyzed by qPCR. Results were normalized to shed oocysts per grams of stool. Error bars were calculated as ±SEM (^∗^*P* < 0.05, ^∗∗^*P* < 0.01, and ^∗∗∗∗^*P* < 0.0001).

All infected mice began shedding oocysts between Days 5 and 7. Stool samples were collected from individual mice, processed and analyzed by qPCR. At Day 10, infected mice treated with PBS and with 100 mg/kg/day of paromomycin were shedding 8.52 × 10^7^ and 1.84 × 10^8^ oocysts per gram of stool (oo/g of stool) respectively (data not shown). Similarly, mice treated with 10 mg/kg/day of miltefosine were shedding 1.94 × 10^8^ oo/g of stool at Day 10 (**Figure 3**). At Days 14 and 30, surviving mice of this group continued to shed considerable levels of oocysts; 1.49 × 10^7^ and 1.5 × 10^7^ oo/g of stool respectively (**Figure 3**).

In mice treated with 40 mg/kg/day of OlPC, oocyst shedding reduction occurred as early as Day 10 with only 7.59 × 10^6^ oo/g of stool which is a significant reduction in comparison with the 10 mg/kg miltefosine group (*P* < 0.01; **Figure 3**). At Day 14, after 10 days of OlPC-treatment, oocyst shedding in this group was even lower with 5.14 × 10^3^ oo/g, representing a 99.96% reduction (4-log reduction) in oocyst shedding. At Day 14, the 40 mg/kg/day OlPC-treated group is also statistically different when compared with the 10 mg/kg miltefosine-treated mice (*P* < 0.05; **Figure 3**). Finally, at Day 30, there was a 100% elimination of oocyst shedding (>7-log reduction) in all mice of the 40 mg/kg/day OlPC-treated group (*P* < 0.0001; **Figure 3**). Light microscopy was used to confirm qPCR results. Indeed, all the samples of 40 mg/kg/day OlPC-treated mice at Day 30 were negative as no oocysts could be visualized under the microscope.

Mice treated with 30 mg/kg/day of OlPC showed a similar tendency in oocyst shedding as the 40 mg/kg/day OlPC-treated group. Effectively, all average oocyst counts were significantly lower when compared with the 10 mg/kg/day miltefosine-treated group (**Figure 3**); at Day 10, the average oocyst shedding was 2.45 × 10^6^ oo/g of stool (*P* < 0.0001), at Day 14, 4.9 × 10^2^ oo/g of stool (*P* < 0.05) and, at Day 30, 13 oo/g of stool (*P* < 0.01). Mice treated with 20 mg/kg/day of OlPC had the least important reduction in oocyst shedding but remained significantly lower than the 10 mg/kg miltefosine-treated mice. In fact, 4.04 × 10^7^, 3.68 × 10^5^, and 5.95 × 10^3^ oo/g of stool, was evaluated in these 20 mg/kg/day OlPC-treated mice at Day 10 (*P* < 0.01), Day 14 (*P* < 0.01), and Day 30 (*P* < 0.05) respectively (**Figure 3**).

Oocyst shedding was not statistically different between mice treated with 30 mg/kg/day and 40 mg/kg/day at Days 10 and 14. The fact that the numerical value of the mean for parasite burden/g of stool for the 40 mg/kg/day treated mice was slightly higher at Days 10 and 14 than the one for 30 mg/kg/day treated mice is most probably due to inter-individual variations in this mouse model.

### OlPC Significantly Reduces Parasite Burden in the Intestines of C57BL/6 IFNγR-KO Mice Infected with *C. parvum*

**FIGURE 4:**
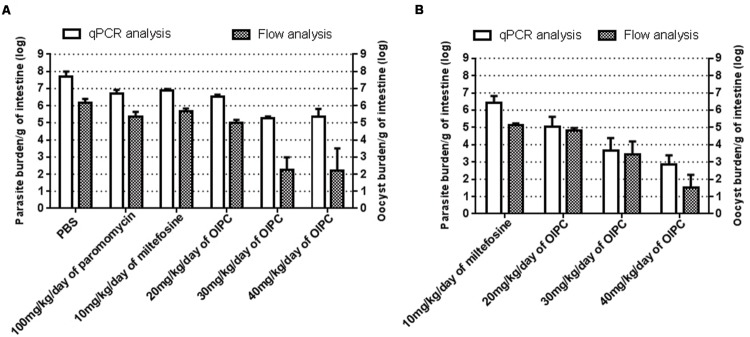
**Parasite and oocyst burden in C57BL/6 IFNγR-KO mice infected with *C. parvum.*** All intestinal samples were analyzed by qPCR represented by the clear bars and by flow cytometry represented by the checkered bars. qPCR results were normalized to parasites per gram of intestines and flow cytometry results were normalized to oocysts per gram of intestines. **(A)** Parasite and oocyst burden in mice at Day 10. **(B)** Parasite and oocyst burden in mice at Day 30. Error bars were calculated as ±SEM.

To quantitate parasite and oocyst burden in the intestines and assess disease progression of infected mice during and after their respective treatments, mice were euthanized and intestines were collected at two time points: Days 10 or 30. Intestinal samples from every mouse were processed individually to purify *C. parvum* parasites, analyzed using qPCR and normalized to parasites per gram of intestines (p/g of intestines). On Day 10, infected control groups revealed massive levels of *C. parvum* in the gut. Mice treated with PBS had the highest parasite burden with 4.77 × 10^7^ p/g of intestines followed by mice treated with 100 mg/kg/day of paromomycin with 5.01 × 10^6^ p/g of intestines (**Figure 4A**). Mice treated with 10 mg/kg/day of miltefosine also had very high parasite burden with 7.94 × 10^6^ p/g of intestines (**Figure 4A**). In mice treated with OlPC, parasite burden was significantly lower compared to the infected PBS control group. After 7 days of treatment (Day 10), 20 mg/kg/day OlPC-treated mice showed a parasite burden of 3.44 × 10^6^ p/g of intestines (*P* < 0.01; **Figure 4A**). In mice treated with 30 mg/kg/day of OlPC the parasite burden was even lower with 1.81 × 10^5^ p/g of intestines (*P* < 0.001) corresponding to a 99.6% (2-log reduction) parasite burden reduction in comparison with the PBS control group (**Figure 4A**). Similarly, mice treated with 40 mg/kg/day of OlPC also revealed a 99.6% reduction in parasite burden with 2.25 × 10^5^ p/g of intestines (*P* < 0.001) when compared to the infected PBS control mice (**Figure 4A**).

By Day 30, 17 days after the completion of the treatments, surviving mice treated with 10 mg/kg miltefosine exhibited a high parasite burden with 2.65 × 10^6^ p/g of intestines (**Figure 4B**). Parasite burden decreased in mice treated with OlPC when compared to the 10 mg/kg miltefosine-treated mice following a dose-dependent response. Indeed, in mice treated with 20 mg/kg/day of OlPC, parasite burden obtained was 1.09 × 10^5^ p/g of intestines (not significant, *P* > 0.05), whereas in mice treated with 30 mg/kg/day and 40 mg/kg/day, parasite burden was 4.66 × 10^3^ p/g of intestines (99.8% parasite burden reduction, *P* < 0.05) and 692 p/g of intestines (99.97% parasite burden reduction, *P* < 0.01) respectively (**Figure 4B**). Additionally, by Day 30 there was an overall reduction in parasite burden in each group compared to their burden at Day 10.

To cross-validate results obtained by qPCR and to confirm the presence of *C. parvum* oocysts in the intestines, flow cytometry was used to quantify the number of oocysts per gram of intestines (oo/g of intestines). Flow cytometry data analysis allowed to identify oocysts by their size and morphology (FSC-A and SSC-A) and to count them for each intestinal sample. In general, total parasite burdens calculated by flow cytometry were lower than those obtained by qPCR suggesting the presence of other stages of the parasite in the intestines that flow cytometry cannot distinguish. However, the results were supportive of those of the qPCR. Mice treated with PBS, 100 mg/kg paromomycin and 10 mg/kg miltefosine showed an oocyst burden of 1.46 × 10^6^ oo/g of intestines, 2.3 × 10^5^ oo/g of intestines and 4.53 × 10^5^ oo/g of intestines respectively (**Figure 4A**). In mice treated with 20 mg/kg/day of OlPC, 9.4 × 10^4^ oo/g of intestines was detected which consisted in a 93.5% decrease in oocyst burden compared to the PBS treated group (*P* < 0.01). The reductions in oocyst burden increase in mice treated with 30 or 40 mg/kg/day of OlPC were only 180 oo/g of intestines (99.98% reduction, *P* < 0.01) and 167 oo/g of intestines (99.98% reduction, *P* < 0.01) respectively (**Figure 4A**). In both cases an average 4-log reduction in oocyst burden was observed when compared to PBS treated mice. In surviving mice treated with 10 mg/kg miltefosine, oocyst burden at Day 30 was 1.4 × 10^5^ oo/g of intestines which is comparable to their oocyst burden at Day 10 (**Figures 4A,B**). At Day 30, at 20 mg/kg/day of OlPC, oocyst levels were slightly lower (6.63 × 10^4^ oo/g of intestines) than mice treated with 10 mg/kg/day of miltefosine, but this difference remained statistically non-significant (52.6% oocyst burden reduction, **Figure 4B**). At 30 mg/kg/day of OlPC, oocyst burden (2.81 × 10^3^ oo/g of intestines) increased compared to the oocyst burden at Day 10 but still represented a 98% oocyst burden reduction compared to the oocyst burden at Day 30 for 10 mg/kg miltefosine-treated mice (**Figure 4B**). Finally, at 40 mg/kg/day of OlPC, mice showed the lowest oocyst burden in their intestines with 34 oo/g of intestine representing a 99.98% oocyst burden reduction when compared to the 10 mg/kg miltefosine-treated mice (*P* < 0.01, **Figure 4B**).

### OlPC Eliminates the Presence of Oocysts in Histological Sections of the Ileum of C57BL/6 IFNγR-KO Mice Infected with *C. parvum*

**FIGURE 5:**
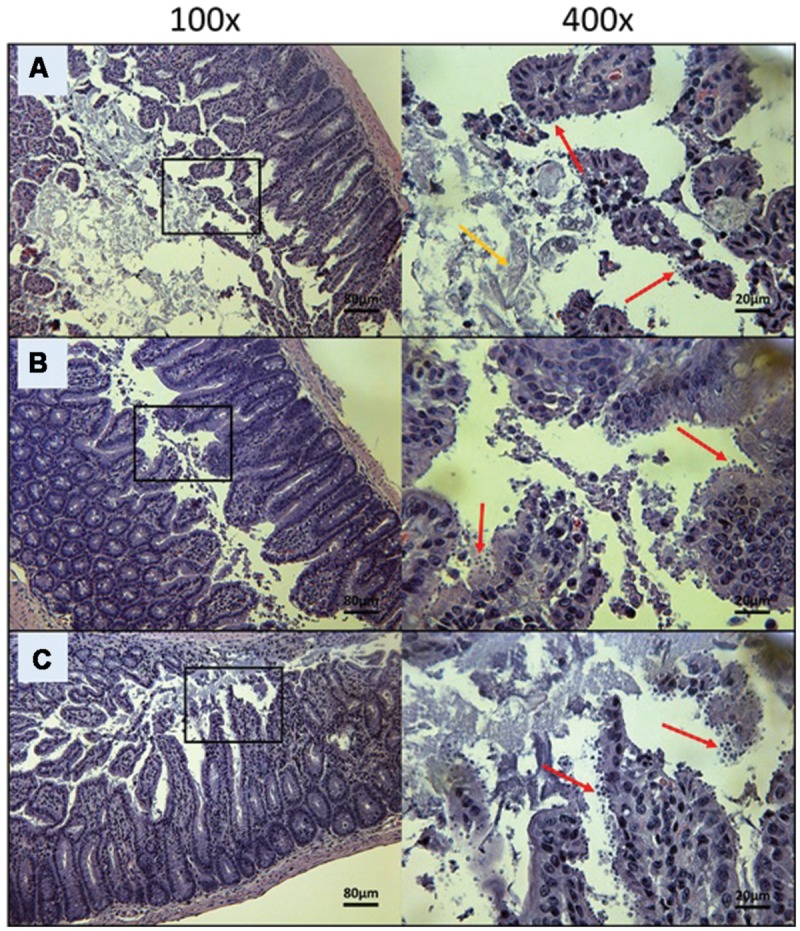
**Day 10 histological sections of the ileum of control C57BL/6 IFNγR-KO mice infected with *C. parvum*.** Representative transverse sections of the ileum stained with H&E showing disease progression. **(A)** Ileum of mice treated with PBS. Formation of fibrin is noticeable (yellow arrow). **(B)** Ileum of mice treated with 100 mg/kg/day of paromomycin. **(C)** Ileum of mice treated with 10 mg/kg/day of miltefosine. **(A–C)** Shows severe epithelial damage and abundance of oocysts. Each group is represented at 100 and 400x magnifications. Red arrows indicate *C. parvum* oocysts.

**FIGURE 6:**
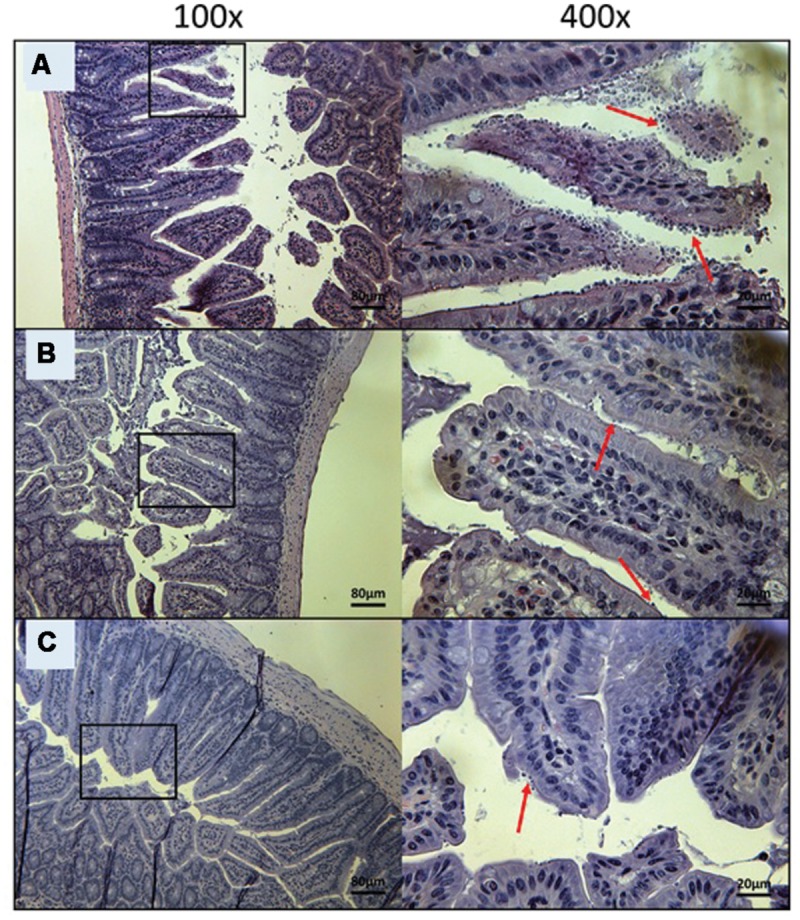
**Day 10 histological sections of the ileum of OlPC-treated C57BL/6 IFNγR-KO mice infected with *C. parvum*.** Representative transverse sections of the ileum stained with H&E showing disease progression. **(A)** Ileum of mice treated with 20 mg/kg/day of OlPC shows significant epithelial damage and abundance of oocysts. **(B)** Ileum of mice treated with 30 mg/kg/day of OlPC. **(C)** Ileum of mice treated with 40 mg/kg/day of OlPC. Sections from **(B,C)** reveal mild gut epithelial damage and rare presence of oocysts. Each group is represented at 100 and 400x magnifications. Red arrows indicate *C. parvum* oocysts.

To visually validate the presence of *C. parvum* life cycle stages in the intestines of infected mice, the ileum was collected from all mice at time of death. Sections from the ileum were prepared and stained (H&E) for histopathology purposes. Ileum sections from mice treated with PBS and 100 mg/kg/day of paromomycin at Day 10 revealed an overwhelming presence of *C. parvum* life cycle stages and severe damage to the epithelium of the intestine (**Figures 5A,B**). Damage to intestinal mucosa includes blunting of the villi, acute inflammation and formation of fibrin (fibrinous hemorrhagic enteritis) resulting from local hemorrhage and clotting (**Figures 5A,B**). The ileum of mice treated with 10 mg/kg/day of miltefosine was as severely damaged and infected as the other controls (**Figure 5C**). At Day 10, *C. parvum* oocysts were also abundantly present in mice treated with 20 mg/kg/day of OlPC and intestinal damage was also present but to a lesser extent than in the infected controls (**Figure 6A**). At the dose of 30 mg/kg/day of OlPC, mild intestinal damage and inflammation were noticeable and *C. parvum* oocysts were scarcely visible (**Figure 6B**). Finally, at 40 mg/kg/day, mice displayed practically no signs of intestinal damage or inflammation and a rare presence of *C. parvum* oocysts at Day 10 (**Figure 6C**). Slides from the ileum of non-infected controls were also prepared to provide a negative reference for comparison (data not shown).

**FIGURE 7:**
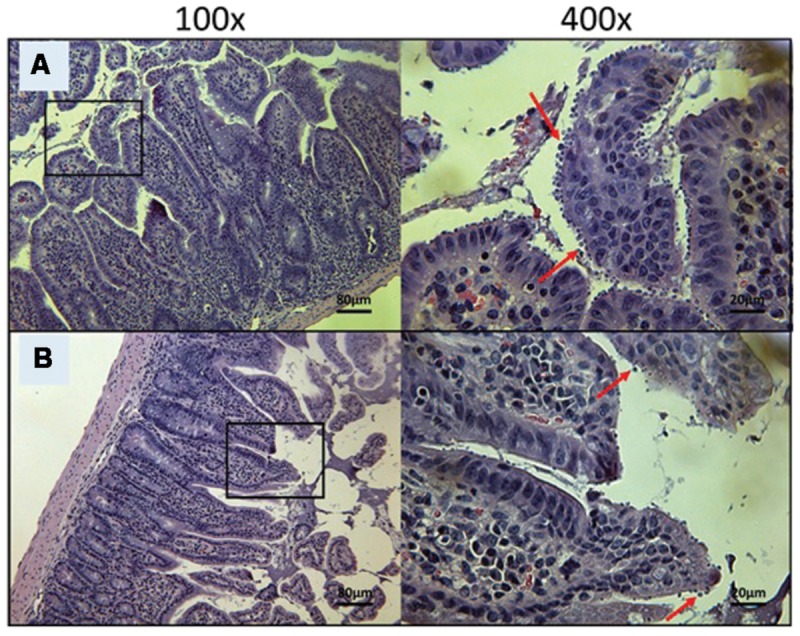
**Day 30 histological sections of the ileum of control and 20 mg/kg/day OlPC-treated C57BL/6 IFNγR-KO mice infected with *C. parvum.*** Representative transverse sections of the ileum stained with H&E showing disease progression. **(A)** Ileum of mice treated with 10 mg/kg/day of miltefosine shows severe epithelial damage and abundance of oocysts. **(B)** Ileum of mice treated with 20 mg/kg/day of OlPC shows some epithelial damage and moderate level of oocysts. Each group is represented at 100 and 400x magnifications. Red arrows indicate *C. parvum* oocysts.

**FIGURE 8:**
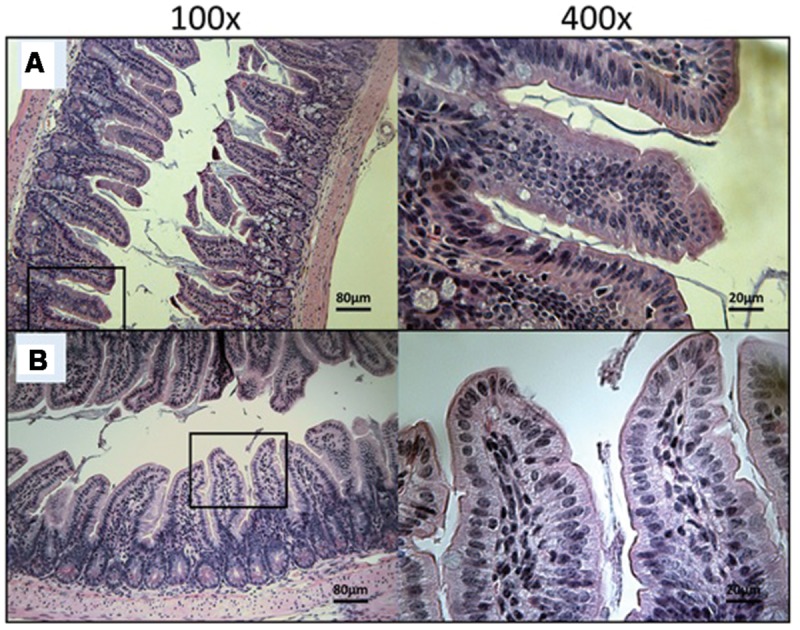
**Day 30 histological sections of the ileum of 30 mg/kg/day OlPC-treated and 40 mg/kg/day OlPC-treated C57BL/6 IFNγR-KO mice infected with *C. parvum.*** Representative transverse sections of the ileum stained with H&E showing disease progression. **(A)** Ileum of mice treated with 30 mg/kg/day of OlPC. Section reveals no gut epithelial damage and an absence of oocysts. **(B)** Ileum of mice treated with 40 mg/kg/day of OlPC presents no sign of epithelial damage and an absence oocysts. Each group is represented at 100 and 400x magnifications.

At Day 30, the surviving mice treated with 10 mg/kg/day of miltefosine revealed an abundant presence of *C. parvum* oocysts and severe damage to the gut epithelium (**Figure 7A**). At the lowest concentration of OlPC, mice displayed a moderate presence of *C. parvum* oocysts and only mild damage to the intestinal epithelium (**Figure 7B**). At 30 and 40 mg/kg/day of OlPC, mice showed a complete absence of *C. parvum* oocysts and a healthy ileum in comparison with controls (**Figures 8A,B**). Sections of ileum, liver and spleen (data not shown) were also processed in uninfected (control) mice treated with 40 mg/kg/day for 10 days and no inflammation or toxicity was noticeable.

### Residual Infectious Oocyst Concentration from Mice Exposed to OlPC is not Sufficient to Cause a New Infection in Naïve C57BL/6 IFNγR-KO Mice

Another experiment was conducted to determine whether there was a sufficient number of infectious oocysts (more than the minimal infectious dose) remaining in the intestines of surviving mice at Days 10 or 30 to transmit the parasite and cause a new infection. To do so, naïve C57BL/6 IFNγR-KO mice were infected with a constant volume of the purified oocysts from mouse intestinal samples coming from the different treatment groups from both Days 10 and 30. In order to determine if naïve mice were infected, stool samples were taken once a week, processed and analyzed by qPCR and light microscopy (**Table 1**). Presence of *C. parvum* oocysts or DNA in the stool of these naïve mice suggests that a fully complete life cycle was achieved by the parasite. Therefore, the concentration of infectious oocysts in the purified intestinal samples of mice from the *in vivo* study would be considered to cause a new infection. Results demonstrated that only the purified intestinal samples from 40 mg/kg/day OlPC-treated mice at Day 30 were not able to successfully transmit a *C. parvum* infection in naïve C57BL/6 IFNγR-KO mice. For these inoculated naïve mice, no oocysts could be seen by light microscopy and no DNA could be detected by qPCR in the stool even by Day 35 (**Table 1**). Additionally, no sign of illness were observed and the histological sections of the ileum revealed a complete absence of oocysts (data not shown). This observation corroborates previous results as 40 mg/kg/day OlPC-treated mice have been shown to have practically cleared the infection at D30. However, naïve C57BL/6 IFNγR-KO mice infected with purified intestinal samples from other treatment groups developed signs of cryptosporidiosis. By Day 17, these inoculated naïve mice exhibited signs of illness (hunched back, weight loss, dehydration, lethargy, and watery stools). Moreover, oocysts could be seen by light microscopy and detected by qPCR (**Table 1**). Histological sections revealed a heavy presence of *C. parvum* oocysts and severe damage to the microvilli (data not shown). These results suggested that the oocysts in the purified intestinal samples of the other groups were sufficient to transmit *C. parvum* to a new host and cause acute cryptosporidiosis.

**Table 1:** Infectivity of purified intestinal samples from the *in vivo* study in naïve C57BL/6 IFNγR-KO mice.

		Day 10^b^		Day 17^b^		Day 35^b^	
Treatment^a^		Microscopy	qPCR	Microscopy	qPCR	Microscopy	qPCR
PBS	Day 10	Yes	Yes	-	-	-	-
	Day 30	-	-	-	-	-	-
100 mg/kg of paromomycin	Day 10	Yes	Yes	-	-	-	-
	Day 30	-	-	-	-	-	-
10 mg/kg of miltefosine	Day 10	Yes	Yes	-	-	-	-
	Day 30	No	No	Yes	Yes	-	-
20 mg/kg of OlPC	Day 10	Yes	Yes	-	-	-	-
	Day 30	No	No	Yes	Yes	-	-
30 mg/kg of OlPC	Day 10	Yes	Yes	-	-	-	-
	Day 30	No	No	Yes	Yes	-	-
40 mg/kg of OlPC	Day 10	No	No	No	Yes	-	-
	Day 30	No	No	No	No	No	No

*^a^Treatment given to mice during the *in vivo* study. Purified intestinal samples from these mice at Days 10 or 30 were used to infect naïve C57BL/6 IFNγR-KO mice.*

*^b^Represents the day post-inoculation that the stool from naïve mice was collected. The presence of *C. parvum* oocysts or its DNA in the stool of naïve mice is scored qualitatively. Dashes represent no available data due to mouse mortality during study: either there was no purified intestinal sample available to inoculate naïve mice (i.e., no PBS-treated mice in the *in vivo* study survived until Day 30) or naïve mice died during rechallenge study (no naïve mice inoculated with purified intestinal samples from mice treated with miltefosine survived until stool collection on Day 35).*

## Discussion

*Cryptosporidium parvum* is a recognized threat to public health (Guerrant, 1997; Feldmann et al., 2002) and has been reported in more than 40 countries in all five continents (Putignani and Menichella, 2010). Many waterborne outbreaks reported in the past decade do not limit *C. parvum* infection to developing countries, but have been reported in Canada, USA, Sweden, and France as a result of tap water contamination (Putignani and Menichella, 2010). On one hand, *Cryptosporidium* causes persistent diarrhea and stunting in young children (Putignani and Menichella, 2010; Bouzid et al., 2013; Richard et al., 2014; Shikani and Weiss, 2014) and, on the other hand, AIDS patients can develop a chronic infection that can be fatal (Chen et al., 2002; Chalmers and Davies, 2010; Bouzid et al., 2013; Mead and Arrowood, 2014). The major issue comes from the lack of efficacious treatment options for cryptosporidial infections (Mead and Arrowood, 2014).

In this present study, we addressed the need for a new efficacious treatment against cryptosporidiosis by testing the compound OlPC, an alkylphosphocholine drug, already in clinical development against leishmaniasis. As a close analog of miltefosine, a drug recently approved by the FDA to treat cutaneous, mucosal and visceral leishmaniasis (FDA, 2014) and to treat primary amebic meningoencephalitis (CDC, 2013), OlPC is suspected to share a similar mode of action by interfering with parasitic lipid biosynthesis and cellular membrane integrity while inducing apoptosis of the parasite (Dorlo et al., 2012). Repurposing OlPC as an anti-cryptosporidiosis drug would have several advantages as the safety of this compound is already reported and pharmacological as well as pharmacokinetics studies are already ongoing (Fortin et al., 2012).

Initial *in vitro* results demonstrated that OlPC efficiently inhibits *C. parvum* infection of HCT-8 cells after 48 h exposure to the drug. OlPC reduced parasite burden by 84% at ≥10 μM and exhibited significant activity down to 10 nM with an EC_50_ of 18.84 nM. We report here that the concentrations used for OlPC and miltefosine to achieve a significant reduction in *C. parvum* infectivity were far lower than the one published in the literature for paromomycin (EC_50_ of 711 μM), the currently used drug against cryptosporidiosis in AIDS patients (Armson et al., 1999; Downey et al., 2008). The toxicity profile displayed by OlPC was also very low with a 40% reduction in host cell viability only at 100 μM and higher. Conversely, it was reported that HCT-8 cells presented signs of toxicity when exposed to miltefosine for 48 h at concentrations as low as 24.5 μM (Shahiduzzaman et al., 2009). Therefore, OlPC surpasses miltefosine in regards to its safety in host cells. Even though many drugs have been shown to successfully inhibit *C. parvum* infection *in vitro*, many of them have failed to inhibit cryptosporidiosis in an animal model (Mead and Arrowood, 2014). Such is the case for monensin (Blagburn et al., 1991) and paromomycin which only started to show efficacy in dexamethasone immunosuppressed mice at very high doses of 1 g/kg/day (Healey et al., 1995). Because the action of the drugs on *C. parvum* parasites is more direct when tested *in vitro* than in an animal model, it is possible that the results from the former does not translate to the latter (Mead and Arrowood, 2014). In fact, many factors influence the effect of drugs *in vivo* on pathogens such as its bioavailability, pharmacodynamics and pharmacokinetics as well as food and drug interactions. Thus, it is important to understand the limitations of the *C. parvum in vitro* model to mimic an *in vivo* model of infection and we must acknowledge *in vitro* results only as informative precursors to further investigations in animal models.

Therefore, to determine whether the *in vitro* activity of OlPC on *C. parvum* could be translated *in vivo*, we decided to use C57BL/6 IFNγR-KO mice, an animal model highly susceptible to *C. parvum* infection (You and Mead, 1998; von Oettingen et al., 2008). Unlike other immunocompromised animal models where a very large *C. parvum* inoculum must be given (such as the dexamethasone (Yang and Healey, 1993; Healey et al., 1995), the SCID (Mead et al., 1995; Tzipori et al., 1995), the neonatal (Downey et al., 2008) or the malnourished models (Costa et al., 2012), C57BL/6 IFNγR-KO mice can consistently develop severe illness with an infectious dose of only 10 oocysts (Yang et al., 2000; von Oettingen et al., 2008). It has also been demonstrated that doses as low as 1000 oocysts were lethal by Days 9–14 (Mead and You, 1998; You and Mead, 1998) and 1500 oocysts lead to 80% death in these mice (Ndao et al., 2013). In our study, mice received 4000 oocysts by oral gavage and began receiving treatment at Day 3 for a period of 10 consecutive days. By increasing the inoculum in these mice, we were able to achieve 100% mortality in PBS treated mice by Day 10 which allowed us to clearly identify the efficacy of the treatment. As early as Day 10, major differences between mice treated with the highest doses of OlPC (30 and 40 mg/kg/day) with other groups could already be seen. Not only did 40 mg/kg/day of OlPC reduce parasite and oocyst burden in the intestinal tract by 99.6 and 99.98% respectively after only 7 days of daily treatment (Day 10), it also allowed 100% of these mice to survive until the experimental endpoint (Day 30; *P* < 0.001). At Day 30, parasite and oocyst burden in the intestines of the 40 mg/kg/day OlPC-treated group reached a 99.97 and 99.98% reduction respectively (*P* < 0.001) in comparison with the 10 mg/kg miltefosine group. These data firmly support that, even after the completion of the treatment with OlPC, mice did not show any sign of relapse in parasite burden or recurrence in clinical symptoms. In addition, a complete elimination of oocyst shedding in the stools was observed and no parasite was noticed by light microscopy of histology section of ileum of 40 mg/kg/day OlPC-treated mice at Day 30. In consequence, it is not surprising that purified intestinal samples from 40 mg/kg/day OlPC-treated mice at D30 were incapable of further transmitting the infection to naïve C57BL/6 IFNγR-KO mice. Together, this validates the hypothesis that OlPC rescued/cured immunocompromised mice from a lethal infection with *C. parvum* and, even if the 40 mg/kg/day OlPC treatment did not completely clear infection at Day 30 (**Figure 4B**), it is not likely that the surviving parasites can cause a recrudescence of infection if mice were not sacrificed at Day 30.

In short, we used a stringent animal model that clearly discriminated if a drug was sufficiently potent to rescue mice from a lethal infection. In this model, paromomycin, given at 100 mg/kg/day for 10 days, failed to rescue mice, whereas in a neonatal model, where the same dosage of paromomycin was given for 6 days, it was able to reduce oocyst burden in the distal colon by 97% and oocyst shedding by 96% (Downey et al., 2008). However, these numbers are not corroborated by clinical cases, where paromomycin treatments are associated with a high probability of relapse in AIDS patients (Hewitt et al., 2000; Cabada and White, 2010). At lower doses, 30 and 20 mg/kg/day of OlPC, treatments were still able to keep 75% of infected mice alive at Day 30, but were not able to eliminate oocyst shedding. Moreover, purified intestinal samples from these mice were capable of transmitting the infection to other naïve C57BL/6 IFNγR-KO mice. This suggests that the dose of these OlPC treatment regimens were not sufficient to eliminate *C. parvum* parasites or to prevent a recurrent infection after the end of the treatment (even though there was a 98% oocyst burden reduction in the intestines by Day 30). Finally, miltefosine was also tested at 10 mg/kg/day for 10 days as a control because of the similar properties and structure it shares with OlPC. Results demonstrated that it had modest, but significant level of activity against *C. parvum* infection since it was able to rescue 50% of infected mice at Day 30. However, the surviving mice were still heavily infected and presented severe signs of illness.

No sign of discomfort or behavioral changes were noted in infected and uninfected mice treated with 40 mg/kg/day of OlPC for 10 days, which is supportive of data previously obtained in a mouse model of leishmaniasis (Fortin et al., 2014). Additionally, sections of the ileum, liver and spleen from these mice showed no signs of toxicity or inflammation associated to OlPC (data not shown).

The present study did not compare miltefosine and OlPC at the same dosages across the entire range. While this may be perceived as a limitation, it was reflective of concerns regarding miltefosine toxicity at higher doses (FDA, 2014).

In summary, the strong activity of OlPC on *C. parvum* parasites *in vitro* was thoroughly supported by the outcome of the animals in our *in vivo* study as OlPC rescues C57BL/6 IFNγR-KO mice from a lethal infection of *C. parvum.* Data obtained by qPCR from the *in vivo* study was not only cross-validated by flow cytometry and light microscopy, but was also confirmed visually by histological sections from mice ileums. Furthermore, OlPC demonstrated, at its highest dose, a lasting effect and mice showed no sign of relapse of infection even 17 days after the end of the treatment. As an analog of miltefosine, we hypothesize that OlPC will have the same mechanism of action on the parasite, that is by causing an irreversible inhibition of the phospholipid biosynthesis pathway leading to the apoptosis in the parasite; further studies will be needed to confirm this hypothesis (Anthony et al., 1999; Dorlo et al., 2012). Together, this establishes OlPC as novel, consistent, sustainable, safe, and potent anti-cryptosporidial treatment option for immunocompromised patients.

## Disclosure

AF works as a consultant for Dafra Pharma R&D.

## Author Contributions

KSD and AER performed *in vitro* experiments, data analysis and manuscript preparation. AER and FVC performed *in vivo* studies in mice. TZDL and AER performed flow cytometry data acquiring and analysis. AM processed stool and intestinal samples from mice. MJA provided *C. parvum* oocysts for experiments. AF provided the OlPC compound and advice on experiment design. MN designed and supervised all experiments. KSD, MJA, AF, and MN improved manuscript to final approved version. All authors reviewed final version of the manuscript and agreed on accuracy.

## Conflict of Interest Statement

The authors declare that the research was conducted in the absence of any commercial or financial relationships that could be construed as a potential conflict of interest.
